# Single-cell transcriptomic reconstruction reveals cell cycle and multi-lineage differentiation defects in *Bcl11a*-deficient hematopoietic stem cells

**DOI:** 10.1186/s13059-015-0739-5

**Published:** 2015-09-21

**Authors:** Jason C. H. Tsang, Yong Yu, Shannon Burke, Florian Buettner, Cui Wang, Aleksandra A. Kolodziejczyk, Sarah A. Teichmann, Liming Lu, Pentao Liu

**Affiliations:** 1 Wellcome Trust Sanger Institute, Wellcome Trust Genome Campus, Hinxton, Cambridgeshire CB10 1SA UK; 2 Wellcome Trust - Medical Research Council Cambridge Stem Cell Institute, University of Cambridge, Tennis Court Road, Cambridge, CB2 1QR UK; 3 EMBL-European Bioinformatics Institute, Wellcome Trust Genome Campus, Hinxton, Cambridgeshire CB10 1SD UK; 4 Shanghai Institute of Immunology, Shanghai Jiao Tong University School of Medicine, Shanghai, 200025 China; 5 Helmholtz Zentrum München - German Research Center for Environmental Health, Institute of Computational Biology, Neuherberg, Germany

## Abstract

**Background:**

Hematopoietic stem cells (HSCs) are a rare cell type with the ability of long-term self-renewal and multipotency to reconstitute all blood lineages. HSCs are typically purified from the bone marrow using cell surface markers. Recent studies have identified significant cellular heterogeneities in the HSC compartment with subsets of HSCs displaying lineage bias. We previously discovered that the transcription factor Bcl11a has critical functions in the lymphoid development of the HSC compartment.

**Results:**

In this report, we employ single-cell transcriptomic analysis to dissect the molecular heterogeneities in HSCs. We profile the transcriptomes of 180 highly purified HSCs (*Bcl11a*^+/+^ and *Bcl11a*^−/−^). Detailed analysis of the RNA-seq data identifies cell cycle activity as the major source of transcriptomic variation in the HSC compartment, which allows reconstruction of HSC cell cycle progression in silico. Single-cell RNA-seq profiling of *Bcl11a*^−/−^ HSCs reveals abnormal proliferative phenotypes. Analysis of lineage gene expression suggests that the *Bcl11a*^−/−^ HSCs are constituted of two distinct myeloerythroid-restricted subpopulations. Remarkably, similar myeloid-restricted cells could also be detected in the wild-type HSC compartment, suggesting selective elimination of lymphoid-competent HSCs after *Bcl11a* deletion. These defects are experimentally validated in serial transplantation experiments where *Bcl11a*^−/−^ HSCs are myeloerythroid-restricted and defective in self-renewal.

**Conclusions:**

Our study demonstrates the power of single-cell transcriptomics in dissecting cellular process and lineage heterogeneities in stem cell compartments, and further reveals the molecular and cellular defects in the *Bcl11a*-deficient HSC compartment.

**Electronic supplementary material:**

The online version of this article (doi:10.1186/s13059-015-0739-5) contains supplementary material, which is available to authorized users.

## Background

Continuous and responsive hematopoiesis is essential for hematopoietic homeostasis throughout the life of a mammalian individual. The classical model of hematopoiesis described this process in a developmental hierarchy, where multipotent hematopoietic stem cells (HSCs) situate at the apex [1]. Purified HSCs are able to provide long-term reconstitution to all blood lineages in transplantation experiments [2–5]. In adult mice, HSCs reside in the bone marrow and remain predominantly mitotically quiescent. Only a small portion of HSCs are cycling to maintain hematopoietic homeostasis or in response to stress and injury [6]. Lymphoid and myeloid blood cells are derived from successive differentiation of respective lineage progenitors with the loss of reconstituting potential upon commitment. HSCs generate multipotent progenitors, which in turn give rise to lineage progenitors, i.e., the common myeloid progenitors (CMPs) and common lymphoid progenitors (CLP). CLPs subsequently produce B/T lymphocytes, whereas CMPs further differentiate to megakaryocyte–eryhroid progenitors (MEPs) and granulocyte–macrophage progenitors (GMPs) to reconstitute erythrocytes, platelets and myeloid cells. Transcription regulators play critical roles in the HSC compartment and direct stem cell differentiation [7, 8]. We have previously reported that the C2H2 zinc finger transcription factor B cell CLL/lymphoma 11A (*Bcl11a*) is essential for both fetal and adult lymphoid development [9, 10]. *Bcl11a* is also highly expressed in the adult HSC compartment, and *Bcl11a*-deficient HSCs fail to contribute to CLP development in vivo [9]. Increasing evidence suggests that the phenotypically defined HSC compartment is heterogeneous and a portion of HSCs display lineage bias or restriction [8, 11–15]. It is possible that the lymphoid defect in *Bcl11a*-deficient HSCs is dependent on its functions in the regulation of lineage-priming heterogeneities in the HSC compartment. However, unbiased transcriptomic dissection of the HSC compartment at the single-cell level was previously hampered by the rarity of HSCs. This technical obstacle has recently been overcome by the advance of RNA-seq technology [16, 17], which allows genome-wide transcriptome analysis to be conducted at the single-cell level.

In this report, we applied a semi-automated microfluidic system to dissect the transcriptomic heterogeneities and reconstruct the cell cycle progression in the Lin^−^ Sca1^+^ Kit^+^ (LSK) CD150^+^48^−^ and Lin^−^ Sca1^+^ Kit^+^ (LSK) CD150^+^48^−^34^−^135^−^ HSC compartments at the single-cell level. These exercises further revealed the molecular and cellular defects in the *Bcl11a*-deficient HSC compartment, which only contains myeloerythroid-restricted progenitor-like cells with substantial self-renewal defects.

## Results

### Single-cell transcriptome profiling of adult HSCs using a microfluidic system

**Fig. 1:**
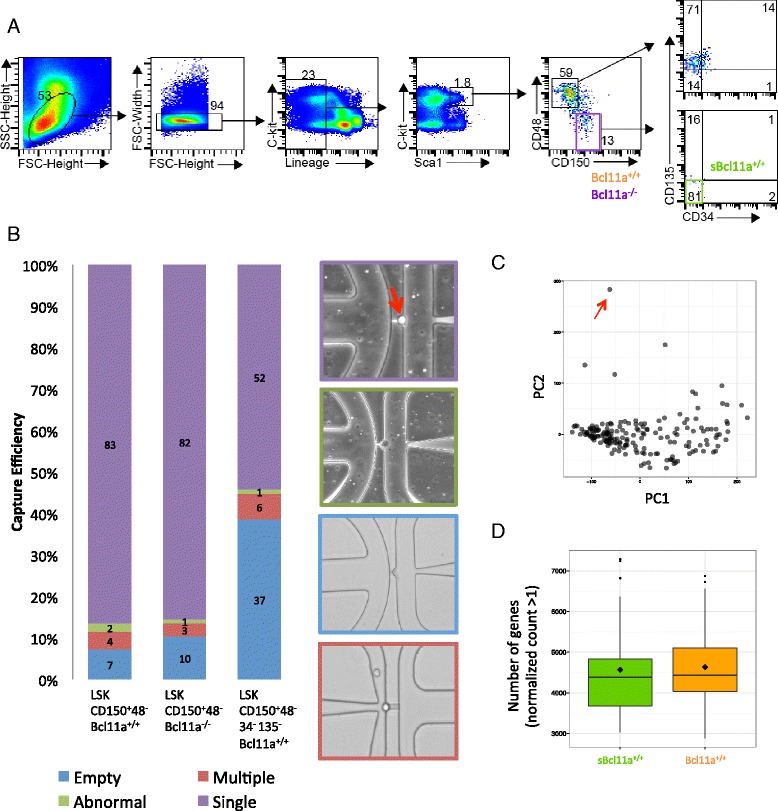
Single-cell transcriptome profiling of mouse HSCs by microfluidic system. **a** Gating strategy for HSC purification. *Bcl11a* ^*+/+*^ and *Bcl11a* ^*−/−*^ HSCs were isolated by sorting markers LSK CD150^+^48^−^ and *sBcl11a* ^*+/+*^ HSCs by markers LSK CD150^+^48^−^34^−^135^−^. Lineage markers used for enrichment included B220, CD19, CD3, CD4, CD8, TCRγδ, TCRβ, NK1.1, CD11b, Gr-1, Ter119. FSC: Forward scatter, SSC: Side scatter. **b** Single-cell capturing efficiency by the C1 AutoPrep microfluidic system and representative microscopic images of individual capture sites. A representative high-quality single HSC at an individual capture site is indicated by the *red arrow*. Representative pictures of poor quality cells, an empty capture site and a multiplet capture site are framed in colors as indicated in the key. **c** Principal component analysis of the transcriptome of all 181 HSCs passed initial computational quality control. One significant outlier from the *Bcl11a* ^*+/+*^ dataset was identified (marked by *red arrow*). It was removed from subsequent analysis. **d** Boxplot comparing the number of genes detected (normalized count >1) in the *sBcl11a* ^*+/+*^ and *Bcl11a* ^*+/+*^ datasets. The two datasets were comparable, despite low sequencing depth of the *sBcl11a* ^*+/+*^ dataset

We employed the Fluidigm microfluidic system coupled with multiplex barcoding to streamline the workflow in cell sorting and cDNA generation. The use of the microfluidic system allowed us to visually confirm the presence of single cells in individual capture sites under the microscope and to filter out debris that may otherwise be mistaken as single cells in flow cytometry. A maximum of 96 cells can be captured and cDNA reverse transcription could be generated in situ in each integrated fluidic circuit. We analyzed the LSK CD150^+^48^−^ HSC compartment (*Bcl11a*^+/+^) from eight *Bcl11a-GFP* reporter conditional knockout mice that we previously described [9] (Fig. 1a). An *IRES-eGFP* cassette was targeted at the 3’ untranslated region of the *Bcl11a* locus, which enabled tracking of *Bcl11a* expression by green fluorescent protein (GFP). The reporter mice have normal HSC development and functions [9]. Additionally, we also purified HSCs (*sBcl11a*^+/+^) with a more stringent sorting scheme (LSK CD150^+^48^−^34^−^135^−^) from ten wild-type C57BL/6 mice (Fig. 1a). Notably, the majority (81 %) of the LSK CD150^+^48^−^ compartment is also LSK CD150^+^48^−^34^−^135^−^, in contrast to the progenitor cell compartment (14 % of LSK CD150^−^48^+^), indicating significant overlap between the sorting schemes. The single-cell capture rate by the microfluidic integrated fluidic circuit was 86.5 % (83/96) and 54.2 % (52/96) for *Bcl11a*^+/+^ and *sBcl11a*^+/+^ HSCs, respectively (Fig. 1b). To investigate the role of *Bcl11a* in the HSC compartment, we used *Bcl11a*^*flox/flox*^; *ROSA26-Cre-ERT2* mice to obtain LSK CD150^+^48^−^ Bcl11a^−/−^ HSCs (*Bcl11a*^*−/−*^) [9]. *Bcl11a* deletion was induced by treating the mice with tamoxifen [9]. One week after tamoxifen treatment, HSCs were purified using fluorescence-activated cell sorting (FACS) from five mice and the single-cell capture rate was 85.4 % (82/96). Cell lysis, cDNA reverse transcription and pre-amplification by PCR were performed and harvested by the C1 Single-Cell Auto Prep system. The sequencing libraries of individual cells of each experiment group (*Bcl11a*^*+/+*^, *sBcl11a*^*+/+*^ or *Bcl11a*^*−/−*^) were then pooled separately and sequenced. After quality control, cells with low numbers of reads (<500,000 in annotated genes), low numbers of detectable genes (<3000 annotated genes), high fractions of mitochondrially encoded transcripts (>10 %) or anomalies seen under microscope were filtered out. We also removed cells showing incomplete *Bcl11a* exon 4 deletion in the *Bcl11a*^*−/−*^ dataset (Additional file 1). In total, 76 *Bcl11a*^*+/+*^ HSCs, 44 *sBcl11a*^*+/+*^ HSCs and 61 *Bcl11a*^*−/−*^ HSCs were further analyzed. The average number of unique counts of genes was 3.16 million (range 1.43–4.52 million) per cell (*Bcl11a*^*+/+*^), 0.89 million (range 0.55–1.71 million) per cell (*sBcl11a*^*+/+*^) and 3.67 million (range 0.54–12.5 million) per cell (*Bcl11a*^*−/−*^). Standardized External RNA Controls Consortium (ERCC) RNA spike-ins were added to the sequencing library to account for the technical variability of the protocol [18]. One significant outlier cell from the *Bcl11a*^*+/+*^ dataset was removed from downstream analysis after principal component analysis (PCA; Fig. 1c). The lower count number from the *sBcl11a*^*+/+*^ dataset is not unexpected due to the lower sequencing depth ("Methods and materials"), but the numbers of genes detected (normalized count >1) between two wild-type datasets after size factor normalization are comparable (Wilcox rank sum test *p* = 0.362) (Fig. 1d).

### Cell cycle activity represents the dominant source of transcriptional heterogeneity in the HSC compartment

**Fig. 2:**
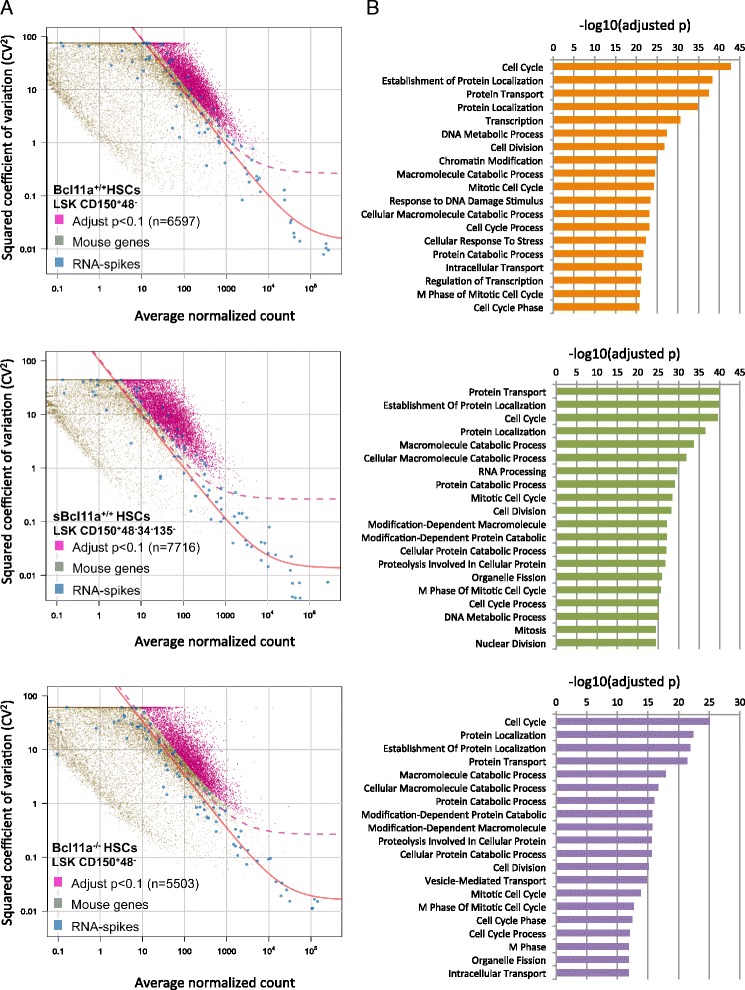
Identification of cell cycle activity as the dominant source of transcriptional heterogeneities in the HSC compartment. **a** Identification of highly variable genes in *Bcl11a* ^*+/+*^ (*upper panel*), *sBcl11a* ^*+/+*^ (*middle panel*) and *Bcl11a* ^*−/−*^ (*lower panel*) HSCs. The expression variability of individual genes measured by the squared coefficient of variation (CV^2^) is plotted against the mean expression level (normalized counts). *Magenta points* indicate mouse genes (*Bcl11a* ^*+/+*^, 6597; *sBcl11a* ^*+/+*^, 7716; *Bcl11a* ^*−/−*^, 5503) showing higher than expected expression variability compared with external RNA spike-ins (*blue*) (adjusted *p* value <0.1). The *red line* is the fitted line of the spike-ins and the *dashed line* marks the margin for genes with 50 % biological CV. **b** The top 20 gene ontology terms enriched in the highly variable genes among *Bcl11a* ^*+/+*^ (*upper panel*), *sBcl11a* ^*+/+*^ (*middle panel*) and *Bcl11a* ^*−/−*^ (*lower panel*) HSCs. The *p* value was adjusted by the Benjamini and Hochberg method for multiple testing

Single-cell transcriptomic analysis allows the detection of gene expression variability between individual cells and identification of cellular subpopulations. Expression variability of a particular gene could come from technical sources (e.g., stochasticity of reverse transcription reaction and library preparation) or genuine biological sources (e.g., differences in cellular states, distinct biological subpopulations and transcription kinetics). It is crucial, therefore, to properly account for the technical variability in single-cell transcriptomic data interpretation. Brennecke et al. [18] recently described a statistical approach to address this problem by the addition of standardized external RNA spike-ins to the sequencing library. The null hypothesis is that the expression variability detected in a particular gene is not different from the technical variability measured by the external RNA spike-ins; thus, genes that display higher than expected variability imply genuine biological fluctuation from possible cellular subgroups. We identified 6,597, 7,716 and 5503 highly variable genes in the *Bcl11a*^*+/+*^, *sBcl11a*^*+/+*^ and *Bcl11a*^*−/−*^ datasets, respectively (Fig. 2a; Additional file 2). Remarkably, gene ontology (GO) term enrichment analysis showed that terms related to cell cycle were significantly over-represented in all three datasets (*p* < 0.0001; Fig. 2b; Additional file 2). This result suggested that cell cycle activity is the dominant source of transcriptomic heterogeneity among HSCs.

### Transcriptional reconstruction of cell cycle progression in the HSC compartment

**Fig. 3:**
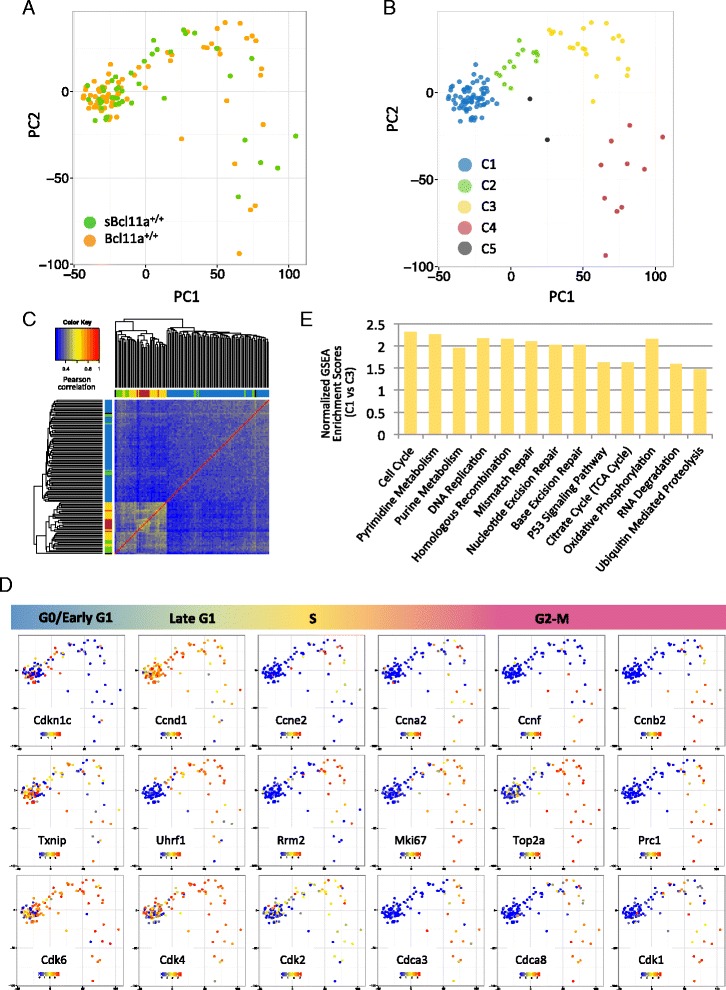
Transcriptomic reconstruction of cell cycle progression by single-cell RNA-seq. **a** PCA of *Bcl11a* ^*+/+*^ HSCs based on the expression of 2212 cell cycle-related genes annotated in the GO, Cyclebase database and Oki et al. [19]. The loading plot of the first two principal components is shown. Each point represents one HSC. **b** Subgrouping of *Bcl11a* ^*+/+*^ HSCs by PCA. The *Bcl11a* ^*+/+*^ HSCs were grouped into five different clusters (C1–5) based on their proximity in the PCA loading plot as in (**a**). **c** Hierarchical clustering and Pearson correlation heatmap of *Bcl11a* ^*+/+*^ HSCs. Correlation between *Bcl11a* ^*+/+*^ HSCs was estimated by the Pearson correlation coefficients. The column and row colors shown above and to the left correspond to the five subgroups by PCA as in (**b**). **d** PCA loading plots of *Bcl11a* ^*+/+*^ HSCs as in (**a**) with expression of selected cell cycle stage-specific genes overlaid. The expression level was calculated as log_10_ (normalized counts +1). **e** The normalized enrichment scores of significantly enriched gene sets (*p* < 0.05, false discovery rate < 0.05) in the C3 cluster compared with the C1 cluster curated by the Kyoto Encyclopedia of Genes and Genomes (KEGG) database. *GSEA* gene set enrichment analysis

To resolve the cell cycle heterogeneities in the HSC compartment, we focused on the expression pattern of cell cycle-associated genes in the merged *Bcl11a*^*+/+*^ HSC dataset (*Bcl11a*^*+/+*^ and *sBcl11a*^*+/+*^). We compiled a list of 2212 cell cycle-associated genes based on the annotations in the GO term “cell cycle”, the Cyclebase database and a recent study on G0/G1 transition by Oki et al. [19] (Additional file 3). PCA was performed to transform the high dimensional expression data into individual linearly uncorrelated principal components. Interestingly, we observed that HSCs displayed a characteristic distribution pattern in the first two principal components (PC1/PC2; Fig. 3a). To aid visualization, we grouped HSCs into five smaller clusters (C1–5) (Fig. 3a) based on their proximity in the PCA plot (Fig. 3b). The grouping is largely consistent with hierarchical clustering (Fig. 3c). To understand if the distribution of the HSCs depicted a cell cycle progression trajectory, we inspected the expression pattern of stage-specific genes based on their annotations in the Cyclebase database and from literature [20]. It appeared that genes situated at different spatial domains of the plot showed clustered expression of stage-specific genes. For instance, the C1 clusters showed high expression of the quiescence regulator of *Cdkn1c* (p57) and G1 arrest factor *Txnip* [21, 22] and lack of the methylation and G1/S entry regulator *Uhrf1* [23] (Fig. 3d). C3 was dominated by S phase-specific genes such as *Rrm2* [24] and DNA replication-licensing factors (e.g., *Mcm2*, *Mcm5*, *Mcm7*), while the C4 cluster expressed high levels of genes involved in cell division (e.g., *Prc1*, *Mki67* (*Ki-67*)) (Fig. 3d). Correspondence of individual clusters to specific cell cycle stages is further supported by the distinct pattern of cyclin and cyclin-dependent kinase expression in specific clusters (Fig. 3d). Cyclins are known to oscillate at specific cell cycle stages when they form complexes with partnering cyclin-dependent kinases to drive cell cycle progression [25]. Cyclin E (*Ccne2*) regulates G1/S transition with CDK2 (*Cdk2*), cyclin A (*Ccna2*) is specifically active in S phase, and cyclin B (*Ccnb2*) functions at G2/M phase transition with CDK1 (*Cdk1*). Cyclin F expression closely followed that of cyclins A and B [26]. Cyclin D (*Ccnd2*) is first expressed in G1 phase and involved in G1/S transition, and its expression is sustained in proliferative cells. The expression levels of specific cyclin genes therefore provided a good molecular marker to define the cell cycle stages of different cell clusters. Concordant to the clustering pattern in PCA, the cyclin expression patterns across clusters C1–4 closely recapitulated the known cyclin oscillation during cell cycle progression (Fig. 3d). This correlation combined with the expression patterns of Cdkn1c and Txnip allowed us to reconstruct a transcriptomically defined cell cycle and assign the cell cycle stage of C1 (72/119 cells) as G0/early G1 phase, C2 (14/119) as late G1 phase, C3 (21/119) as S phase and C4 (10/119) as G2/M phase (Fig. 3b, d). The status of the C5 clusters is undetermined due to the low number of cells. Interestingly, the fraction of HSCs classified as G0/early G1 phase in both the *Bcl11a*^*+/*+^ (28 %, 54/75) and *sBcl11a*^*+/+*^ (27.3 %, 32/44) datasets are similar, consistent with our finding that the sorting schemes of LSK CD150^+^48^−^ and LSK CD150^+^48^−^34^−^135^−^ are mostly overlapping (Fig. 1a).

We then applied this classification to explore the molecular differences between the “G0/early G1 phase” C1 and “S phase” C3 clusters. We performed gene set enrichment analysis (GSEA) on gene sets curated in the Kyoto Encyclopedia of Genes and Genomes (KEGG) database. Gene sets related to “cell cycle” and “DNA replication” are significantly enriched as expected (Fig. 3e). In addition, we also detected significant enrichment of gene sets involved in DNA damage repair such as “mismatch repair” and “nucleotide/base excision repair”. This finding is in accordance with recent reports that quiescent adult HSCs accumulate DNA damage and this damage is repaired upon entry into the cell cycle [27–29]. Similarly, enrichment of “p53 signaling pathway” is also detected in the proliferative C3 cluster, consistent with its role in the orchestration of DNA damage repair [30]. Furthermore, gene set enrichment of “oxidative phosphorylation” and “citrate cycle” in the C3 cluster is supported by the observation that quiescent HSCs undergo a glycolytic to aerobic metabolic transition upon activation [31]. Interestingly, gene sets associated with “ubiquitin-mediated proteolysis” is also significantly enriched, affirming the role of the ubiquitin proteasome system in the regulation of HSC cell cycle regulation [32]. These results suggest that transcriptomic cell cycle reconstruction by single-cell RNA-seq data permits direct comparison of the biological characteristics of HSCs at different cell cycle stages in silico.

### Acute *Bcl11a* deletion in the HSC compartment alters cell cycle progression

**Fig. 4:**
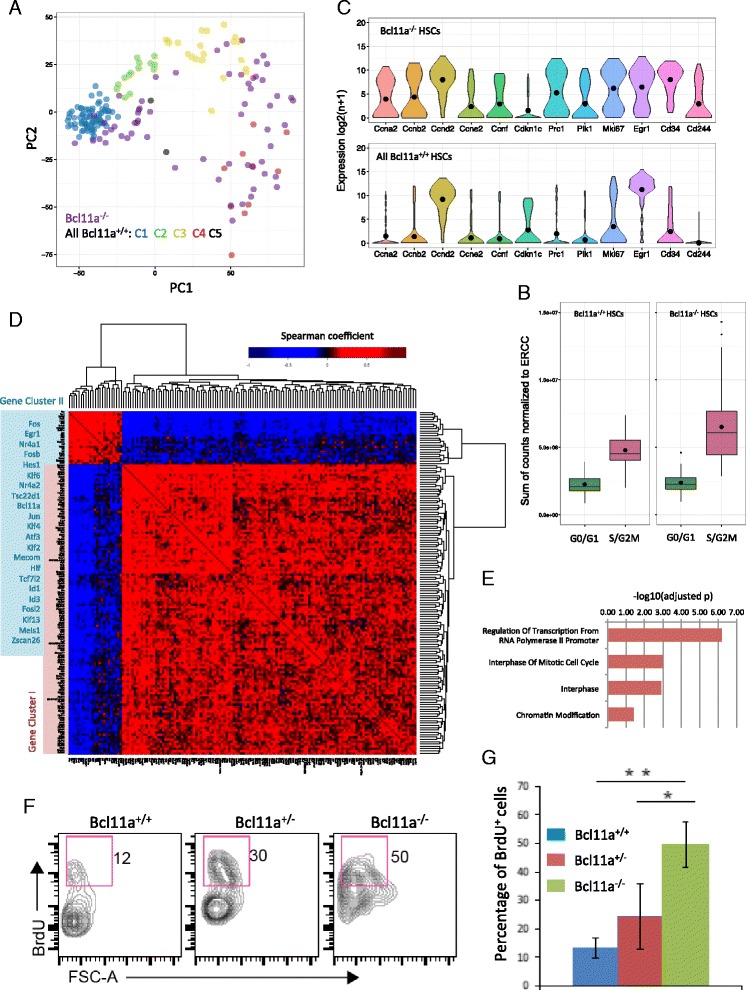
*Bcl11a*-deficient HSCs showed significant proliferative changes in the HSC compartment. **a** Distribution of *Bcl11a* ^*−/−*^ HSCs (*purple*) and cell cycle reconstructed-*Bcl11a* ^*+/+*^ HSC clusters (C1–C5) in a PCA loading plot of the first two principal components. **b** Boxplots comparing the transcriptional activity of G0/G1 stage (C1 and C2) and S/G2/M stage (C3 and C4) HSCs in the *Bcl11a* ^*+/+*^ and *Bcl11a* ^*−/−*^ datasets, estimated by the total number of read counts normalized by ERCC size factor per cell. **c** Violin plots of gene expression of selected cyclin genes, progenitor markers and cell cycle stage-associated genes in *Bcl11a* ^*+/+*^ and *Bcl11a* ^*−/−*^ HSCs. The *black dots* represent the mean expression for each gene. **d** Heatmap showing expression correlations of selected transcription regulators in the HSC compartment. Correlation coefficient was calculated by Spearman correlation coefficient and clustering was performed by complete linkage. Gene correlation cluster II (*blue*) is magnified. **e** Enriched GO terms in gene correlation cluster I [*red* in (**d**)]. **f** Validation of cell cycle changes in *Bcl11a* ^*−/−*^ HSCs by 5-bromodeoxyuridine (BrdU) staining. The *purple box* marks the BrdU^+^ fraction in the HSC compartment of different genotypes. *FSC-A: Forward scatter area*. **g** The dose-dependent changes in BrdU^+^ cell number in the HSC compartment in different *Bcl11a* genotypes. HSCs were harvested and sorted from adult mouse bone marrow 5 days after tamoxifen-induced Bcl11a ablation. *p < 0.05, **p < 0.01, n = 3 mice for each group. *Bcl11a* ^*+/−*^, CreERT2; *Bcl11a* ^*+/flox*^ (treated with tamoxifen); *Bcl11a* ^*−/−*^, CreERT2; *Bcl11a* ^*flox/flox*^ (treated with tamoxifen). The error bar represented mean ± 1 standard deviation

We next examined the HSC compartment when *Bcl11a* was deleted. Notably, the distribution of *Bcl11a*^*−/−*^ HSCs in PCA closely mimicked that of merged *Bcl11a*^*+/+*^ HSCs in terms of cell cycle-related genes (Fig. 4a). Nevertheless, there were more *Bcl11a*^*−/−*^ cells (59.0 %, 36/61 cells) in the space occupied by the proliferative C3 and C4 clusters of the control *Bcl11a*^*+/+*^ HSCs (26.1 %, 31/119) (Fig. 4a), suggesting that *Bcl11a* deficiency is associated with increased proliferation in the HSC compartment. Moreover, we observed a significant increase of transcription activity in the proliferating *Bcl11a*^*−/−*^ HSCs compared with controls, estimated by the total number of ERCC-normalized counts per cell (Fig. 4b). This is consistent with a previous report that transcription rate periodically increases from G1 to S/G2/M phase [33] and suggests that *Bcl11a*^*−/−*^ HSCs are more transcriptionally active. There was also evident general up-regulation of cyclin genes and down-regulation of the quiescence regulator *Cdkn1c* (p57) and G2/M markers such as *Prc1*, *Plk1* and *Mki67* (*Ki-67*) [20] (Fig. 4c). Notably, genes encoding HSC activation and progenitor markers such as *Cd34* and *Cd244* were also elevated [34, 35]. Moreover, examination of the gene expression correlation of transcription factors revealed that there were two anti-correlative gene clusters (Fig. 4d, cluster I in red and II in blue) in the HSC compartment. Cluster I genes were enriched for GO cell cycle terms like “interphase”, suggesting their actions in cell cycle regulation (Fig. 4e). Meanwhile, the expression of *Bcl11a* in cluster II correlated with multiple known HSC quiescence regulators such as *Egr1*[36], *Fos* [37], *Mecom* [38], *Meis1* [39], *Hlf* [40], and *Nr4a1* [41] (Fig. 4d). Signaling mediators important for HSC quiescence, such as Notch signaling (*Hes1*) [42], Wnt signaling (*Tcf7l2*) [42] and TGFβ signaling (*Id1* and *Id3*) [43] can also be found in cluster II. These suggest that *Bcl11a* may cooperate with these regulators in maintaining HSC quiescence. To confirm the prediction that *Bcl11a*^*−/−*^ HSCs have reduced quiescence, we performed in vivo 5-bromodeoxyuridine (BrdU) staining for cell cycle profiling. As predicted, significantly higher numbers of BrdU^+^ cells were found in either the *Bcl11a*^*+/−*^ or *Bcl11a*^*−/−*^ HSC compartment compared with the control (Fig. 4f, g). Furthermore, *Bcl11a*^*−/−*^ HSC compartment had significantly more proliferative cells compared with haploinsufficient *Bcl11a*^*+/−*^ HSCs, indicating a dose-dependent requirement for *Bcl11a*. These transcriptomic and experimental results demonstrate a cell cycle defect in the *Bcl11a*^*−/−*^ HSC compartment.

### *Bcl11a*-deficient HSCs have defects in long-term self-renewal potential

**Fig. 5:**
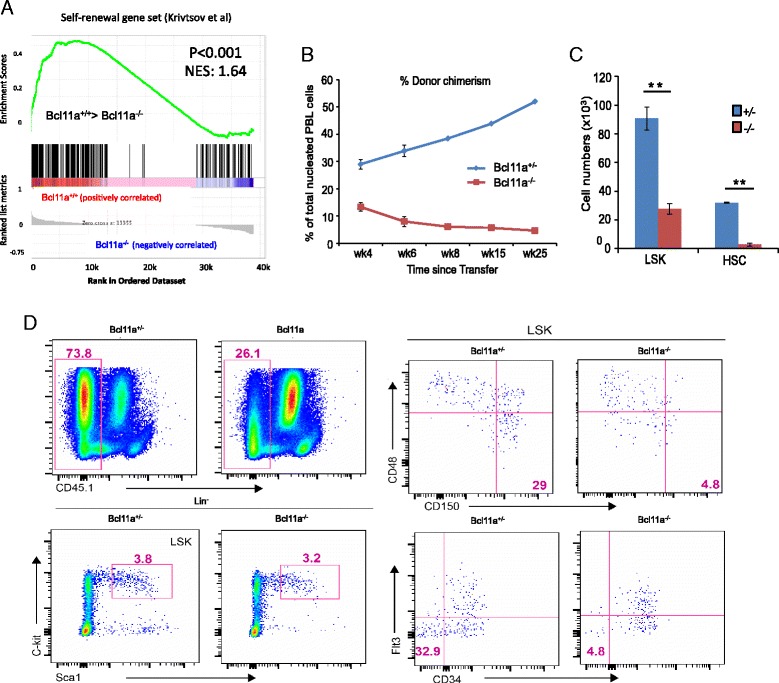
*Bcl11a*-deficient HSCs show self-renewal defects. **a** Gene set enrichment analysis showing significant depletion of the self-renewal gene signature in *Bcl11a* ^*−/−*^ HSCs (*p* < 0.001). The normalized enrichment score (*NES*) of 1.64 indicates significantly higher self-renewal gene expression in the *Bcl11a* ^*+/+*^ HSCs compared with *Bcl11a* ^*−/−*^ HSCs. **b** Percentage of donor cells in total nucleated peripheral blood (*PBL*) cells along with time after transfer. We injected 2000 *Bcl11a* ^*+/−*^ or *Bcl11a* ^*−/−*^ LSKs (CD45.1^−^) with 1.0 × 10^6^ bone marrow (BM) cells (CD45.1^+^) into sublethally irradiated recipient mice (CD45.1^+^). **c** Comparison of the number of donor LSK cells and HSCs in secondary recipient mice 18 weeks post-secondary transfer; *p < 0.05, **p < 0.01. **d** FACS dot plot of donor *Bcl11a* ^*+/−*^ and *Bcl11a* ^*−/−*^ HSCs (LSK CD150^+^48^−^ and LSK CD34^−^135(Flt3)^−^) in secondary recipient mice. BM cells were analyzed 18 weeks post-secondary transfer. *Bcl11a* ^*+/−*^, CreERT2; *Bcl11a* ^*+/flox*^ (treated with tamoxifen); *Bcl11a* ^*−/−*^, *CreERT2*; *Bcl11a* ^*flox/flox*^ (treated with tamoxifen). In panels (**b**) and (**c**), at least three mice were used for each time point or each cell type in independent experiments. The error bar represented mean ± 1 standard deviation

The cell cycle defect in *Bcl11a*-deficient HSCs prompted us to examine their self-renewal potential. We first examined the expression of the self-renewal gene signature by GSEA. We retrieved the signature gene set described by Krivtsov et al. [44] as associated with HSC and leukemic stem cell self-renewal (Additional file 3). We detected a highly significant reduction of expression in *Bcl11a*^*−/−*^ HSCs (Fig. 5a). To experimentally test this observation, we transplanted purified *Bcl11a*^*−/−*^ or *Bcl11a*^*+/−*^ LSKs (CD45.1^−^) to sublethally irradiated CD45.1^+^ recipient mice. Donor contribution in the peripheral blood was examined at different time points after the adoptive transfer. *Bcl11a*^*−/−*^ LSKs had substantially lower capacity to generate hematopoietic progenies, and contributed much less efficiently in the recipients than the *Bcl11a*^*+/−*^ LSKs (Fig. 5b). Furthermore, the secondary recipient mice had drastically decreased *Bcl11a*^*−/−*^ HSCs, both percentage-wise and total cell numbers (Fig. 5c, d), which was further confirmed by using an alternative HSC sorting scheme (LSK CD34^−^135^−^) (Fig. 5d).

### *Bcl11a* deletion eliminated lymphoid-competent HSCs and resulted in expansion of two myeloerythroid-restricted subpopulations in the HSC compartment

We have previously demonstrated experimentally that *Bcl11a* is essential in the generation of lymphoid progenitors, including lymphoid-primed multipotent progenitors and CLPs, but its effects on HSCs at the single-cell level were unclear [9]. We thus attempted to interrogate the effect of Bcl11a deficiency on the lineage multipotency of HSCs with our single-cell transcriptomic dataset.

**Fig. 6:**
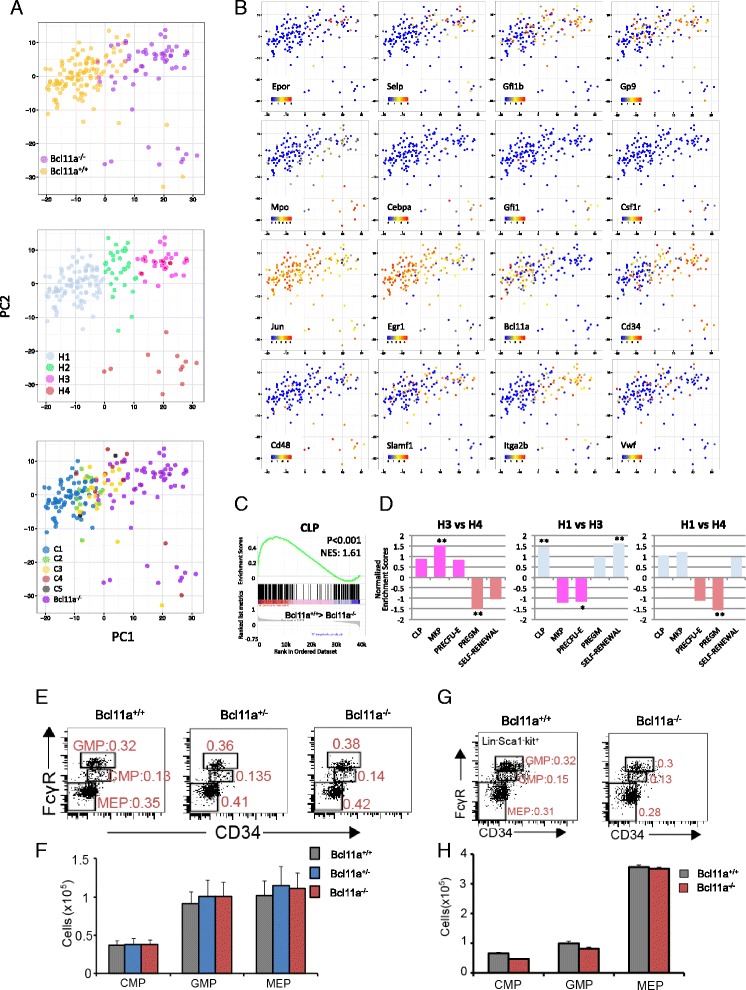
The *Bcl11a*-deficient HSC compartment contained two distinct myeloerythroid subpopulations and showed myeloerythroid-restriction in lineage reconstitution. **a** PCA loading plot of HSCs based on their expression of hematopoietic genes. *Upper panel*: the HSCs were labeled based on their corresponding genotypes. *Middle panel*: HSCs were grouped into four different subgroups and colored based on their locations in the loading plot. *Lower panel*: HSCs were labeled based on their cell cycle stages defined in Fig. 3b. **b** PCA loading plot of HSCs with expression of selected hematopoietic genes overlaid. The expression level is calculated as log_10_(Normalized counts + 1). **c** GSEA showing significant depletion of the common lymphoid progenitor signature in *Bcl11a* ^*−/−*^ HSCs (*p* < 0.001). The normalized enrichment score (*NES*) of 1.61 indicates significantly higher lymphoid signature in the *Bcl11a* ^*+/+*^ HSCs compared with *Bcl11a* ^*−/−*^ HSCs. **d** Normalized enrichment scores of different lineage progenitor signatures in different subgroup pair-wise comparisons. *: p <0.05, **: p <0.01 **e** Analysis of the number of bone marrow (BM) CMPs, GMPs and MEPs with different genotypes by flow cytometry one week after tamoxifen injection. Numbers refer to percentages in total BM nucleated cells. Lin^−^ BM cells were analyzed and *n* = 4 mice for each genotype. **f** Comparison of the numbers of myeloerythroid progenitors with different genotypes as in (**e**). **g** Analysis of the number of BM CMPs, GMPs and MEPs in primary recipient by flow cytometry 8 weeks after LSK and helper BM cells transplantation from donors (CD45.1^−^) with different genotypes. Numbers refer to percentages of progenitors in total BM nucleated cells. **h** Comparison of the cell numbers of myeloerythroid progenitors in different genotypes as in (**g**). Cells were harvested from the two femurs of each mouse (*n* = 4/genotype). The error bar represented mean ± 1 standard deviation

We compiled a list of genes from the annotation “hematopoiesis and lymphoid organ development” in the GO database and the literature (Additional file 3) and studied their expression in HSCs with PCA. Annotated hematopoietic genes also involved in cell cycle activity were removed from the compilation to reduce their effect on heterogeneity dissection. Notably, significant overlap of *Bcl11a*^*+/+*^ and *sBcl11a*^*+/+*^ HSCs can be observed in the PCA (Fig. 6a, upper panel). The *Bcl11a*^*−/−*^ HSCs are segregated into two uneven subpopulations, with the larger subpopulation partially overlapping the merged *Bcl11a*^*+/+*^ (*Bcl11a*^*+/+*^ and *sBcl11a*^*+/+*^) HSCs. The smaller and more scattered *Bcl11a*^*−/−*^ HSC subpopulation clusters with few *sBcl11a*^*+/+*^ HSCs at the right lower quadrant of the loading plot of the first two principal components (Fig. 6a, upper panel).

To aid visualization and downstream comparison, we again grouped the HSCs shown in Fig. 6a into four subgroups (H1–4) based on their locations on the loading plot (Fig. 6a, middle panel). Intriguingly, the expression pattern of hematopoietic genes pointed to a clustered expression of markers associated with specific lineages (Fig. 6b). For instance, expression of mega-erythroid genes such as *Epor*, *Selp*, *Gp9*, and *Gfi1b* [45, 46] were clustered in subgroups H2 and H3, while myelopoietic genes such as *Mpo*, *Cebpa* [47], *Gfi1* [48], and *Csf1r* were concentrated at the H4 subgroup. Interestingly, the known antagonistic regulation between *Gfi1* and *Gfi1b* is recapitulated by the reciprocal exclusion of the two regulators in the H3 and H4 subgroups [49, 50]. Compared with the H1 subgroup, the H3 and H4 subgroups showed reduced expression of lymphoid and HSC activation regulators such as *Egr1*, *Bcl11a*, and *Jun*, but prevalent expression of progenitor markers like *Cd34* and *Cd48* (Fig. 6b).

Overlaying the cell cycle staging information on the PCA plot of hematopoietic lineage genes (Fig. 6a, lower panel), we found that most of the cells in the H1 subgroup belong to the C1 (G0/early G1 phase) and C2 (late G1 phase) clusters. Wild-type HSCs which clustered with *Bcl11a*^*−/−*^ HSCs in the H4 subgroup belong to either C3 (S phase) or C4 (G2/M phase) (Fig. 6a, lower panel).

We then performed GSEA to further compare lineage progenitor signature enrichment between the different subgroups (Fig. 6c, d). The signature gene sets were retrieved from Sanjuan-Pla et al. [13] (Additional file 3) and comparison between *Bcl11a*^*+/+*^ and *Bcl11a*^*−/−*^ HSCs showed an expected depletion of the lymphoid signature, consistent with our previous report [9] (Fig. 6c). In addition, the H1 subgroup is enriched with the lymphoid and self-renewal signature, while the H3 and H4 subgroups are enriched with the erythroid and granulocyte-macrophage signatures, respectively (Fig. 6d). Further HSC transplantation experiments also confirmed that *Bcl11a*^*−/−*^ HSCs, which dominated the H3 and H4 subgroups, are myeloid-restricted, and that the myeloid progenitor compartments (CMPs, GMPs and MEPs) were not affected in both acute *Bcl11a* deletion mice and primary recipients after *Bcl11a* deletion (Fig. 6e–h).

Collectively, a trajectory of gradual loss of lymphoid and self-renewal potential can be discerned as wild-type HSCs become activated and proliferative when transiting from the lymphoid-competent H1 subgroup to the mega-erythroid-restricted H3 subgroup or the granulocyte-macrophage-restricted progenitor-like H4 subgroup. More importantly, the existence of *sBcl11a*^*+/+*^ HSCs in the H4 subgroup suggest that myeloid-restricted cells similar to *Bcl11a*^*−/−*^ HSCs have already existed in the wild-type HSC compartment. The *Bcl11a*^*−/−*^ HSCs likely come from expansion or activation of existing myeloid-restricted HSCs in the wild-type compartment after the elimination of lymphoid-competent HSCs by *Bcl11a* deletion.

## Discussion

The hematopoietic system has been an area of intense research because of its paramount clinical potential. The discovery of specific surface markers for purification of HSCs in the adult mouse model allowed us to study the key biological properties of self-renewal and lineage commitment. Recently, increasing evidence from clonal transplantation assays supports the phenotypically defined HSC compartment as being functionally heterogeneous, with subpopulations of platelet-primed or myeloid-restricted HSCs [13, 14]. In this study, we have profiled the transcriptomes of 180 HSCs in the mouse adult HSC compartment and studied the transcriptome structure and the role of *Bcl11a* in the HSC compartment. We show that single-cell transcriptomics could be used to reconstruct biological processes, dissect cellular subpopulations in the HSC compartment, and predict stem cell behavior, such as self-renewal capacity and differentiation potential in silico.

Reconstruction of cell cycle progression at the single-cell level provides a new avenue to study the molecular characteristics of quiescent and proliferative HSCs without additional staining or sorting. This advantage is illustrated by our demonstration of the direct comparison of quiescent and proliferative HSCs defined in the reconstruction by GSEA. Reported features of proliferative HSCs, such as activation of the DNA damage repair pathway and transition to oxidative metabolism, can be clearly identified from such an exercise.We noted that the fraction of proliferative cells classified by the transcriptomic approach is higher than the estimation by conventional BrdU staining in this study (~27 % versus 12 %). Others have reported an even lower fraction (<2 %) of cycling cells with alternative DNA content staining and *Ki67* expression [35]. The discrepancy is not, in fact, unexpected; instead, the transcriptomic approach may provide a more sensitive staging as cell cycle progression requires further translational and post-translational regulation. Changes in the transcriptome are therefore one of the earliest events in entry to the cell cycle. We further extended the use of the reconstruction in predicting cell cycle phenotype in the *Bcl11a*^*−/−*^ HSC compartment. Comparison of *Bcl11a*^*−/−*^ and the control datasets predicted reduction of quiescence in the *Bcl11a*^*−/−*^ HSC compartment, which was experimentally confirmed by BrdU staining. Gene expression correlation analysis revealed a correlative expression pattern of *Bcl11a* with multiple known quiescence regulators and signaling mediators. The analysis provided a basis for future mechanistic dissection of *Bcll1a* in the regulation of HSC self-renewal and quiescence.

We have previously shown that *Bcl11a*^*−/−*^ HSCs are deficient in lymphoid reconstitution [9]. This defect could be caused by blockage of lymphoid commitment at the HSC level or selective elimination of lymphoid-competent HSCs. The possibility to dissect subpopulations using single-cell RNA-seq data provides a new avenue to test these hypotheses.

Segregation of HSCs on the basis of hematopoietic gene expression by PCA revealed an interesting separation of *Bcl11a*^*−/−*^ and *Bcl11a*^*+/+*^ HSC expression patterns with partial overlap (Fig. 6a, upper panel). To better dissect their hematopoietic characteristics, we subdivided all the HSCs sequenced in this study into four groups (H1–4) based on their location proximity. The H1 subgroup overwhelmingly consisted of *Bcl11a*^*+/+*^ HSCs. GSEA of the lineage signature showed that they are enriched with the self-renewal signature and lymphoid potential. Overlaying the PCA plot with cell cycle stage information, we found that the lymphoid-competent H1 HSCs contained all the G0/G1 *Bcl11a*^*+/+*^ HSCs (Fig. 6a, lower panel). In contrast, the H2 subgroups contained both *Bcl11a*^*−/−*^ and *Bcl11a*^*+/+*^ HSCs and the *Bcl11a*^*+/+*^ HSCs in H2 are predominantly proliferative. Few proliferative *Bcl11a*^*+/+*^ HSCs are also present in the H4 subgroup clustering with *Bcl11a*^*−/−*^ HSCs. Compared with the H1 lymphoid-competent subgroup, the H3 and H4 subgroups showed marked enrichment of MEP and GMP progenitor signatures (Fig. 6d) and high expression of activation markers such as *Cd48* and *Cd34*. The computational exercises and experimental data indicate that, firstly, the lymphoid-competent H1 subgroup is selectively depleted after *Bcl11a* deletion. Secondly, the dominance of G0/G1 phase HSCs and the self-renewal signature in the H1 subgroup compared with the H2 subgroup is associated with lymphoid competency and HSC self-renewal. Thirdly, the presence of mixed myeloid-restricted *Bcl11a*^*−/−*^ and *Bcl11a*^*+/+*^ HSCs in the H2 and H4 subgroups suggests the pre-existence of myeloid-restricted *Bcl11a*^*−/−*^*-*like HSCs in the wild-type compartment. Fourthly, the clear MEP- and GMP-like signature distinction in the H3 and H4 subgroups suggests that the myeloerythroid-restricted *Bcl11a*^*−/−*^ HSC compartment is constituted of two distinct progenitor-like subpopulations. Finally, the close relationship of the H3 and H4 subgroups with the H2 subgroup suggests that they may arise from activation and expansion of the latter subpopulations after *Bcl11a* deletion. Based on this evidence, we propose that *Bcl11a* serves two roles in the HSC compartment: in the maintenance of lymphoid-competent HSCs and in the regulation of HSC self-renewal. Deletion of *Bcl11a* selectively eliminated lymphoid-competent HSCs, causing the lymphoid deficiency in the *Bcl11a*^*−/−*^ compartment. The myeloerythroid restrictive phenotype of *Bcl11a*^*−/−*^ HSCs is contributed by the residual surviving myelo-restricted subpopulations in the HSC compartment, which may have limited self-renewal potential.

The detection of *Bcl11a*^*−/−*^-like wild-type HSCs (H2 and H4 subgroups) in the wild-type compartment in our dataset raises interesting questions about their resemblance to the recently reported myeloid-restricted repopulating progenitors in the myeloid bypass model proposed by Yamamoto et al. [14]. In that study, HSCs were purified with a variant sorting scheme (LSK CD150^+^34^−^41^+^), which selectively enriched myeloid-biased HSCs [5, 11, 12, 14]. Notably, *Itga2b* (CD41) was also found to be expressed predominantly in the H2 and H3 clusters in our dataset, supporting the mega-erythroid signature enrichment in these cells (Fig. 6b). Nonetheless, we did not observe clustered expression of *Vwf* in our dataset, which is suggested to mark platelet-primed primitive HSCs in mice [13]. The transcriptomic relationship of these variant HSC compartments with the *Bcl11a*^*−/−*^ HSCs thus warrants further investigation.

## Conclusions

We have successfully applied single-cell transcriptomic analysis in reconstructing the cell cycle process and dissecting the molecular heterogeneities of the rare HSC population. Our data reveal the diversity of lineage-biased subpopulations and the effect of *Bcl11a* in the maintenance of the HSC compartment. *Bcl11a* is essential in the maintenance of lymphoid-competent HSC survival and self-renewal.

## Materials and methods

### Mice

*Bcl11a*^*flox/flox*^*Rosa26*^*CreERT2/CreERT2*^ and *Bcl11a*^*eGFP/eGFP*^ mice were previously reported [9]. F1 hybrid wild-type mice from C57BL/6 CD45.1^+^ crossed with 129S5 mice were used as recipients for transplantation of bone marrow or LSK cells. All mice used were from colonies maintained at the Research Support Facility of the Wellcome Trust Sanger Institute. Housing and breeding of mice and experimental procedures were according to the UK 1986 Animals Scientific Procedure Act and the Animal Welfare and Ethical Review Body of the Wellcome Trust Sanger Institute.

### Flow cytometry

Single-cell suspensions were incubated with purified anti-CD16/32 (clone 93) for 10 min on ice to block Fc receptors. Fluorochrome- or biotin-labeled monoclonal antibodies (clones denoted in parentheses) against B220 (RA3-6B2), CD19 (1D3), CD3ɛ (145-2C11), CD4 (RM4-5), CD8 α (53–6.7), TCRβ (B20.6), NK1.1 (PK136), CD11b (M1/70), Gr1 (RB6-8C5), Ter119 (TER-119), Sca1 (D7), c-kit (2B8), CD48 (HM48-1), CD150 (TC15-12 F 12.2), CD34 (RAM34), and Flt3 (A2F10) were purchased from BD Biosciences, BioLegend or eBioscience. Cells were stained with antibodies on ice for 20 min before washing. Cells were run on a Fortessa (BD Biosciences) or MoFlo XDP (BD Biosciences) and analyzed by Flowjo (Tree Star). For HSC sorting, Lin^−^ cells were enriched by Lineage Cell Depletion Kit (Miltenyi Biotec) before antibody staining.

### Cell-cycle analysis

Mice were given a single intraperitoneal injection of (BrdU (100 μg g^−1^ mouse weight) and then maintained on 0.8 mg/ml BrdU in the drinking water for 14 or 20 h before being euthanized. HSCs were double FACS-sorted from mouse bone marrow. BrdU incorporation was measured by flow cytometry using an APC BrdU Flow Kit (BD Pharmingen) according to the manufacturer’s instructions.

### In vivo transplantation assay

Transplantation assays were performed using the CD45.1/CD45.2 congenic mouse system. The total nucleated bone marrow cells or LSK cells were purified from bone marrow of the flox/flox mice and the control mice (+/flox) treated with tamoxifen for 4–5 days. The bone marrow cells (1–5 × 10^5^) or sorted LSK cells (2000 cells) were injected with helper CD45.1^+^ bone marrow cells (2 × 10^5^ cells) into lethally irradiated (2 × 500 cGy) recipient mice (CD45.1^+^) via the tail vein. For the second bone marrow transplantation, bone marrow cells (0.2–1 × 10^6^) were obtained from recipient mice 16 weeks after first transplantation, and transplanted into a second set of lethally irradiated (2 × 500 cGy) CD45.1^+^ recipient mice.

### Single-cell RNA-seq library preparation

Two thousands FACS-sorted *Bcl11a*^*+/+*^ and *Bcl11a*^*−/−*^ HSCs pooled from eight and five mice, respectively, were loaded onto two separate 5–10 μm C1 Single-Cell Auto Prep integrated fluidic circuits (Fluidigm) and cell capture was performed according to the manufacturer’s protocol. Similarly, 1000 *sBcl11a*^*+/+*^ HSCs from 10 mice were loaded. Individual capture sites were inspected under light microscope to confirm the presence of single cells. The locations of empty capture sites and sites containing multiple cells or burst cells were noted for downstream quality control during data analysis. The lysis and reverse transcription mixes were then prepared with the SMARTer PCR cDNA Synthesis kit (Clontech) and the Advantage 2 PCR kit (Clontech) according to Fluidigm recommendations. The ERCC Spike-In Control Mix (1.0 μl in a 1:1000 dilution; Ambion) was added to the lysis mix to allow control of technical variation of the library preparation protocol. Lysis and cDNA reverse transcription and PCR were performed and cDNA harvested by the C1 Single-Cell Auto Prep system according to the manufacturer’s settings. The success of cDNA preparation was confirmed by optimal DNA signal detected by a 2100 Bioanalyzer with High-sensitivity DNA chip (Agilent). Multiplex sequencing libraries were then prepared using the Nextera DNA Sample Preparation Kit and the Nextera Index Kit (Illumina) according to the recommendation in the C1 Single-Cell Auto Prep manual. The libraries of individual cells of each experiment group (*Bcl11a*^*+/+*^/*Bcl11a*^*−/−*^) were then pooled and sequenced on four lanes of a HiSeq 2500 (Illumina) to generate 100-bp paired-end reads through the Wellcome Trust Sanger Institute in-house sequencing pipeline. For the *sBcl11a*^*+/+*^ HSC library, samples from empty capture sites, abnormal cells or multiplets detected by microscopy were removed before library construction and were similarly sequenced on two lanes of a HiSeq 2500 (Illumina). The raw sequencing data can be found in the European Nucleotide Archive (sudy ERP010829).

### Read alignment and quality control of dataset

Paired-end reads were aligned to the *Mus musculus* genome (Ensembl GRCm38.75 assembly) by GSNAP (version 2013-08-14) with the following parameter settings: ‘gsnap -A sam -B 5 -t 8 -n 1 -Q --nofails’ [51]. The quantification of expression was performed by the htseq-count module from the HTSeq package [52] with gene annotation from GTF files (Ensembl GRCm38.75 assembly) with parameter “–s no” in intersection-nonempty mode [53]. Cells with less than 500,000 counts in annotated features, expression of less than 3000 genes or a high percentage (>10 %) of counts mapping to mitochondrial genes were removed from subsequent analysis. Cells with reads detected on the *Bcl11a* locus chr11:24163146–24165166 were removed from the *Bcl11a*^*−/−*^ dataset as they represent incomplete exon 4 deletion after tamoxifen induction. Significant outliers in PCA based on the whole transcriptome were removed from the merged dataset of *Bcl11a*^*+/+*^, *sBcl11a*^*+/+*^ and *Bcl11a*^*−/−*^ HSCs (Fig. 1d). Cells with anomalies, empty capture sites, or multiple cells at capture sites were removed prior to sequencing in the *sBcl11a*^*+/+*^ dataset. The quality control summary is detailed in Additional file 1.

### Detection of highly variable genes and statistical analysis

The details of the statistical model for testing highly variable genes have been described by Brennecke et al. [18]. We set the minimal biological dispersion parameter at 0.5. PCA was performed with function prcomp() in the *stats* package of R (version 3.0.2 — “Frisbee Sailing”) based on log_2_ transformed count matrix by log_2_(n + 1).

### GO enrichment analysis

GO term enrichment analysis was performed by DAVID Bioinformatics Resources 6.7 [54] with the default mouse genome as background [55].

### Gene set enrichment analysis

GSEA was performed with the javaGSEA application (version 2.0.14) available online [56] with default settings [57]. The lineage-specific gene sets (MkP, PreCFU-E, PreGM, PreMEGE, CLP) were retrieved from a previously published dataset by Sanjuan-Pla et al. [13]. The self-renewal signature gene set was retrieved from Krivtsov et al. [44]. The gene identifiers were remapped by BioMart [58] to official Ensembl gene symbols. A normalized count matrix from DESeq2 was supplied as the input expression dataset. Enrichment is considered significant if the false discovery rate is below 0.1 and the nominal *p* value is below 0.05.

### Statistical analysis

Experimental data are shown as mean and standard deviation. All statistical analyses were either conducted with Prism (GraphPad) or as specified in relevant sections.

## Additional files

Additional file 1: Table S1. Quality control summary of the *Bcl11a* ^*+/+*^, *sBcl11a* ^*+/+*^ and *Bcl11a* ^*−/−*^ HSC dataset. (XLSX 64 kb) Additional file 2: Table S2. The list of highly variable genes in the *Bcl11a* ^*+/+*^, *sBcl11a* ^*+/+*^ and *Bcl11a* ^*−/−*^ HSC dataset and the gene ontology term (biological processes) enrichment analysis results. (XLSX 3257 kb) Additional file 3: Table S3. The list of annotated cell cycle genes and hematopoietic regulators used for PCA and the gene sets used for GSEA in this study. (XLSX 126 kb)

## Abbreviations

BrdU: 5-bromodeoxyuridine
CLP: common lymphoid progenitor
CMP: common myeloid progenitor
ERCC: External RNA Controls Consortium
FACS: fluorescence-activated cell sorting
GFP: green fluorescent protein
GMP: granulocyte–macrophage progenitor
GO: gene ontology
GSEA: gene set enrichment analysis
HSC: hematopoietic stem cell
LSK: Lin^−^ Sca1^+^ Kit^+^
MEP: megakaryocyte–erythroid progenitor
PCA: principal component analysis

## References

[1] Orkin SH, Zon LI (2008). Hematopoiesis: an evolving paradigm for stem cell biology. Cell..

[2] Spangrude GJ, Heimfeld S, Weissman IL (1988). Purification and characterization of mouse hematopoietic stem cells. Science..

[3] Ogawa M, Matsuzaki Y, Nishikawa S, Hayashi S, Kunisada T, Sudo T (1991). Expression and function of c-kit in hemopoietic progenitor cells. J Exp Med..

[4] Christensen JL, Weissman IL (2001). Flk-2 is a marker in hematopoietic stem cell differentiation: a simple method to isolate long-term stem cells. Proc Natl Acad Sci U S A..

[5] Kiel MJ, Yilmaz OH, Iwashita T, Yilmaz OH, Terhorst C, Morrison SJ (2005). SLAM family receptors distinguish hematopoietic stem and progenitor cells and reveal endothelial niches for stem cells. Cell..

[6] Kiel MJ, He S, Ashkenazi R, Gentry SN, Teta M, Kushner JA (2007). Haematopoietic stem cells do not asymmetrically segregate chromosomes or retain BrdU. Nature..

[7] Gottgens B (2015). Regulatory network control of blood stem cells. Blood..

[8] Eaves CJ (2015). Hematopoietic stem cells: concepts, definitions, and the new reality. Blood..

[9] Yu Y, Wang J, Khaled W, Burke S, Li P, Chen X (2012). Bcl11a is essential for lymphoid development and negatively regulates p53. J Exp Med..

[10] Liu P, Keller JR, Ortiz M, Tessarollo L, Rachel RA, Nakamura T (2003). Bcl11a is essential for normal lymphoid development. Nat Immunol..

[11] Morita Y, Ema H, Nakauchi H (2010). Heterogeneity and hierarchy within the most primitive hematopoietic stem cell compartment. J Exp Med..

[12] Gekas C, Graf T (2013). CD41 expression marks myeloid-biased adult hematopoietic stem cells and increases with age. Blood..

[13] Sanjuan-Pla A, Macaulay IC, Jensen CT, Woll PS, Luis TC, Mead A (2013). Platelet-biased stem cells reside at the apex of the haematopoietic stem-cell hierarchy. Nature..

[14] Yamamoto R, Morita Y, Ooehara J, Hamanaka S, Onodera M, Rudolph KL (2013). Clonal analysis unveils self-renewing lineage-restricted progenitors generated directly from hematopoietic stem cells. Cell..

[15] Benz C, Copley MR, Kent DG, Wohrer S, Cortes A, Aghaeepour N (2012). Hematopoietic stem cell subtypes expand differentially during development and display distinct lymphopoietic programs. Cell Stem Cell..

[16] Saliba AE, Westermann AJ, Gorski SA, Vogel J (2014). Single-cell RNA-seq: advances and future challenges. Nucleic Acids Res..

[17] Kolodziejczyk AA, Kim JK, Svensson V, Marioni JC, Teichmann SA (2015). The technology and biology of single-cell RNA sequencing. Mol Cell..

[18] Brennecke P, Anders S, Kim JK, Kolodziejczyk AA, Zhang X, Proserpio V (2013). Accounting for technical noise in single-cell RNA-seq experiments. Nat Methods..

[19] Oki T, Nishimura K, Kitaura J, Togami K, Maehara A, Izawa K (2014). A novel cell-cycle-indicator, mVenus-p27K-, identifies quiescent cells and visualizes G0-G1 transition. Sci Rep..

[20] Santos A, Wernersson R, Jensen LJ (2015). Cyclebase 3.0: a multi-organism database on cell-cycle regulation and phenotypes. Nucleic Acids Res.

[21] Matsumoto A, Takeishi S, Kanie T, Susaki E, Onoyama I, Tateishi Y (2011). p57 is required for quiescence and maintenance of adult hematopoietic stem cells. Cell Stem Cell..

[22] Zou P, Yoshihara H, Hosokawa K, Tai I, Shinmyozu K, Tsukahara F (2011). p57(Kip2) and p27(Kip1) cooperate to maintain hematopoietic stem cell quiescence through interactions with Hsc70. Cell Stem Cell..

[23] Bonapace IM, Latella L, Papait R, Nicassio F, Sacco A, Muto M (2002). Np95 is regulated by E1A during mitotic reactivation of terminally differentiated cells and is essential for S phase entry. J Cell Biol..

[24] Engstrom Y, Eriksson S, Jildevik I, Skog S, Thelander L, Tribukait B (1985). Cell cycle-dependent expression of mammalian ribonucleotide reductase. Differential regulation of the two subunits. J Biol Chem.

[25] Satyanarayana A, Kaldis P (2009). Mammalian cell-cycle regulation: several Cdks, numerous cyclins and diverse compensatory mechanisms. Oncogene..

[26] Bai C, Richman R, Elledge SJ (1994). Human cyclin F. EMBO J..

[27] Beerman I, Seita J, Inlay MA, Weissman IL, Rossi DJ (2014). Quiescent hematopoietic stem cells accumulate DNA damage during aging that is repaired upon entry into cell cycle. Cell Stem Cell..

[28] Flach J, Bakker ST, Mohrin M, Conroy PC, Pietras EM, Reynaud D (2014). Replication stress is a potent driver of functional decline in ageing haematopoietic stem cells. Nature..

[29] Walter D, Lier A, Geiselhart A, Thalheimer FB, Huntscha S, Sobotta MC (2015). Exit from dormancy provokes DNA-damage-induced attrition in haematopoietic stem cells. Nature..

[30] Asai T, Liu Y, Bae N, Nimer SD (2011). The p53 tumor suppressor protein regulates hematopoietic stem cell fate. J Cell Physiol..

[31] Suda T, Takubo K, Semenza GL (2011). Metabolic regulation of hematopoietic stem cells in the hypoxic niche. Cell Stem Cell..

[32] Moran-Crusio K, Reavie LB, Aifantis I (2012). Regulation of hematopoietic stem cell fate by the ubiquitin proteasome system. Trends Immunol..

[33] Zopf CJ, Quinn K, Zeidman J, Maheshri N (2013). Cell-cycle dependence of transcription dominates noise in gene expression. PLoS Comput Biol..

[34] Oguro H, Ding L, Morrison SJ (2013). SLAM family markers resolve functionally distinct subpopulations of hematopoietic stem cells and multipotent progenitors. Cell Stem Cell..

[35] Wilson A, Laurenti E, Oser G, van der Wath RC, Blanco-Bose W, Jaworski M (2008). Hematopoietic stem cells reversibly switch from dormancy to self-renewal during homeostasis and repair. Cell..

[36] Min IM, Pietramaggiori G, Kim FS, Passegue E, Stevenson KE, Wagers AJ (2008). The transcription factor EGR1 controls both the proliferation and localization of hematopoietic stem cells. Cell Stem Cell..

[37] Venezia TA, Merchant AA, Ramos CA, Whitehouse NL, Young AS, Shaw CA (2004). Molecular signatures of proliferation and quiescence in hematopoietic stem cells. PLoS Biol..

[38] Zhang Y, Stehling-Sun S, Lezon-Geyda K, Juneja SC, Coillard L, Chatterjee G (2011). PR-domain-containing Mds1-Evi1 is critical for long-term hematopoietic stem cell function. Blood..

[39] Unnisa Z, Clark JP, Roychoudhury J, Thomas E, Tessarollo L, Copeland NG (2012). Meis1 preserves hematopoietic stem cells in mice by limiting oxidative stress. Blood..

[40] Gazit R, Garrison BS, Rao TN, Shay T, Costello J, Ericson J (2013). Transcriptome analysis identifies regulators of hematopoietic stem and progenitor cells. Stem Cell Rep..

[41] Land RH, Rayne AK, Vanderbeck AN, Barlowe TS, Manjunath S, Gross M (2015). The orphan nuclear receptor NR4A1 specifies a distinct subpopulation of quiescent myeloid-biased long-term HSCs. Stem Cells..

[42] Duncan AW, Rattis FM, DiMascio LN, Congdon KL, Pazianos G, Zhao C (2005). Integration of Notch and Wnt signaling in hematopoietic stem cell maintenance. Nat Immunol..

[43] Perry SS, Zhao Y, Nie L, Cochrane SW, Huang Z, Sun XH (2007). Id1, but not Id3, directs long-term repopulating hematopoietic stem-cell maintenance. Blood..

[44] Krivtsov AV, Twomey D, Feng Z, Stubbs MC, Wang Y, Faber J (2006). Transformation from committed progenitor to leukaemia stem cell initiated by MLL-AF9. Nature..

[45] Osawa M, Yamaguchi T, Nakamura Y, Kaneko S, Onodera M, Sawada K (2002). Erythroid expansion mediated by the Gfi-1B zinc finger protein: role in normal hematopoiesis. Blood..

[46] Saleque S, Cameron S, Orkin SH (2002). The zinc-finger proto-oncogene Gfi-1b is essential for development of the erythroid and megakaryocytic lineages. Genes Dev..

[47] Zhang DE, Zhang P, Wang ND, Hetherington CJ, Darlington GJ, Tenen DG (1997). Absence of granulocyte colony-stimulating factor signaling and neutrophil development in CCAAT enhancer binding protein alpha-deficient mice. Proc Natl Acad Sci U S A..

[48] Karsunky H, Zeng H, Schmidt T, Zevnik B, Kluge R, Schmid KW (2002). Inflammatory reactions and severe neutropenia in mice lacking the transcriptional repressor Gfi1. Nat Genet..

[49] Vassen L, Fiolka K, Mahlmann S, Moroy T (2005). Direct transcriptional repression of the genes encoding the zinc-finger proteins Gfi1b and Gfi1 by Gfi1b. Nucleic Acids Res..

[50] Moignard V, Macaulay IC, Swiers G, Buettner F, Schutte J, Calero-Nieto FJ (2013). Characterization of transcriptional networks in blood stem and progenitor cells using high-throughput single-cell gene expression analysis. Nat Cell Biol..

[51] Wu TD, Nacu S (2010). Fast and SNP-tolerant detection of complex variants and splicing in short reads. Bioinformatics.

[52] HTSeq. http://www-huber.embl.de/users/anders/HTSeq/.

[53] Anders S, Pyl PT, Huber W (2015). HTSeq-a Python framework to work with high-throughput sequencing data. Bioinformatics..

[54] DAVID. http://david.abcc.ncifcrf.gov/home.jsp.

[55] Huang DW, Sherman BT, Tan Q, Kir J, Liu D, Bryant D (2007). DAVID Bioinformatics Resources: expanded annotation database and novel algorithms to better extract biology from large gene lists. Nucleic Acids Res..

[56] javaGSEA. http://www.broadinstitute.org/gsea/downloads.jsp.

[57] Subramanian A, Tamayo P, Mootha VK, Mukherjee S, Ebert BL, Gillette MA (2005). Gene set enrichment analysis: a knowledge-based approach for interpreting genome-wide expression profiles. Proc Natl Acad Sci U S A..

[58] BioMart. http://www.ensembl.org/biomart/martview/

